# Prevalence and associated factors of multi-ethnic adolescent idiopathic scoliosis in Longlin, Southwestern China: a school-based cross-sectional study

**DOI:** 10.3389/fpubh.2025.1729509

**Published:** 2025-12-15

**Authors:** Chengxiang Hu, Baocheng Lin, Fengtao Li, Zhixing Li, Xiaozhuan Chen

**Affiliations:** 1 Department of Joints and Soft Tissue Injury, The Fourth Clinical Medical College of Guangzhou University of Chinese Medicine, Shenzhen, Guangdong, China; 2 Rehabilitation Medicine Department, People's Hospital of Longlin, Longlin Various Nationalities Autonomous County, Baise, Guangxi, China

**Keywords:** adolescent idiopathic scoliosis, prevalence, associated factors, cross-sectional study, multi-ethnic region

## Abstract

**Objective:**

To determine the prevalence of adolescent idiopathic scoliosis (AIS) and its associated factors among multi-ethnic schoolchildren in Southwestern China.

**Methods:**

A cross-sectional school-based study was conducted involving 22,814 students aged 10–18 years. Screening comprised the Adams forward bend test; positives underwent radiographic confirmation (Cobb angle ≥10°). Demographic and behavioral data were collected via questionnaires. Multivariate logistic regression identified independent associated factors.

**Results:**

The screening positive rate was 3.83%, and the confirmed AIS prevalence rate was 1.37%. While no significant ethnic difference existed in screening positive rates (*p* = 0.078), the confirmed prevalence rates differed (*p* = 0.029), being relatively higher in Yi (1.52%) and GeLao (1.54%) adolescents. Girls showed a significantly higher screening positive rate than boys (4.64% vs. 3.12%, *p* < 0.001), but no statistically significant gender difference was found in the confirmed prevalence rates (*p* = 0.157). Notably, the positive predictive value was higher in boys (40.90%) than girls (31.72%). Multivariate analysis revealed several factors independently associated with AIS, including abnormal BMI, lack of desk/chair adjustment, poor sleep posture, physical inactivity, improper reading/writing posture, excessive electronic device use, insufficient outdoor activity, inadequate sleep, and frequent sweet consumption.

**Conclusion:**

The AIS prevalence in this multi-ethnic region aligns with global figures. School screening coupled with health interventions targeting modifiable associated factors is essential for AIS prevention and control. Comprehensive health promotion and early intervention targeting the identified significant factors should be an important future direction for AIS prevention.

## 1 Introduction

Scoliosis is a disease characterized by three-dimensional torsion and deformity of the spine, manifesting in the coronal plane as a lateral curvature with one or more segments deviating from the body's midline; it is typically diagnosed based on a Cobb angle of ≥10° (1, 2). Adolescent Idiopathic Scoliosis (AIS) is the predominant type of scoliosis, accounting for approximately 80% of all cases and predominantly affecting adolescents aged 10–18 years (3–5). AIS not only affects the patient's appearance but can also lead to low back pain, psychological distress, and in severe cases, restricted respiratory function and mobility impairment, imposing a significant burden on families and society (1, 2). Furthermore, the perceived association between poor posture in adolescents and AIS often raises widespread concern and anxiety among patients and their parents (6–8). Therefore, implementing early screening is crucial for the timely detection of AIS, enabling effective intervention and delaying disease progression.

The period from 9 to 13 years old typically represents the peak height velocity (PHV) stage in adolescents, during which the progression of scoliosis is most pronounced (9). Compared to the complex conditions of adult scoliosis, children and adolescent populations are more suitable for screening, and consequently, most domestic and international screening efforts focus on this age group. The global prevalence of AIS is approximately 1.34%, ranging from 0.4% to 2.5% in Asia (6), and about 1% to 3% among individuals aged 10–16 years in the United States (10, 11). Studies in China indicate that the peak prevalence of AIS in individuals aged 4–20 years is concentrated between 13 and 15 years (12).

Numerous studies have indicated a gender difference in AIS prevalence, with rates generally higher in females than in males, at a ratio ranging from 1.5 to 2.5 (12–18). For instance, a screening study covering individuals aged 4–20 years in China reported prevalence rates of 0.87% in males and 1.22% in females, a statistically significant difference (12). However, some studies report contrasting trends, such as one in the Jammu region of India where the prevalence was lower in females (0.31%) than in males (0.88%) (19), suggesting that the clinical presentation of AIS may be influenced by various factors, e.g., genetics, height, body mass index (BMI), and ethnicity (20, 21). A screening study conducted in Tianzhu Tibetan Autonomous County, which is a high-altitude, multi-ethnic region with relatively underdeveloped resources, revealed ethnic disparities in scoliosis prevalence and the angle of trunk rotation, with Han adolescents exhibiting a higher prevalence than their Tibetan minority counterparts. This work addresses a critical epidemiological gap in Western China and elucidates the collective influence of geographic, ethnic, and socioeconomic factors on spinal development, underscoring the value of this unique setting for such research (22). These findings suggest that ethnic background may be one of the factors associated with AIS risk, likely interacting with diverse genetic predispositions, nutritional status, and physical activity patterns.

Studies have shown that for skeletally immature adolescents, especially those with milder curves, non-surgical interventions such as bracing and scoliosis-specific exercises can effectively slow curve progression and reduce the need for surgery (23, 24). Schools, being settings with high concentrations of adolescents, offer convenient venues for large-scale screening, which is why most scoliosis screening programs, both internationally and domestically, are school-based (25–28). *Management Measures for Health Examinations of Primary and Secondary School Students* (2021 Edition, China) explicitly mandates annual health check-ups in primary and secondary schools, providing an institutional foundation for centralized AIS screening. Therefore, utilizing schools as the screening venue facilitates the early diagnosis of AIS and can reduce the need for surgical intervention. However, school screening also faces challenges such as inefficient referral mechanisms (29). It is recommended to incorporate professional collaboration with hospitals during the screening process, establishing a “school organization—hospital support” cooperative model. This ensures the professionalism of screening while facilitating subsequent follow-up and management for those with abnormal screening results.

Based on this, our study targets in-school students at the critical PHV stage, aiming to investigate the prevalence and influencing factors of AIS among adolescents in a multi-ethnic region of Southwestern China, thereby providing a scientific basis for formulating student physical fitness monitoring and health intervention strategies in this region.

## 2 Objects and methods

### 2.1 Study design and participants

**Figure 1:**
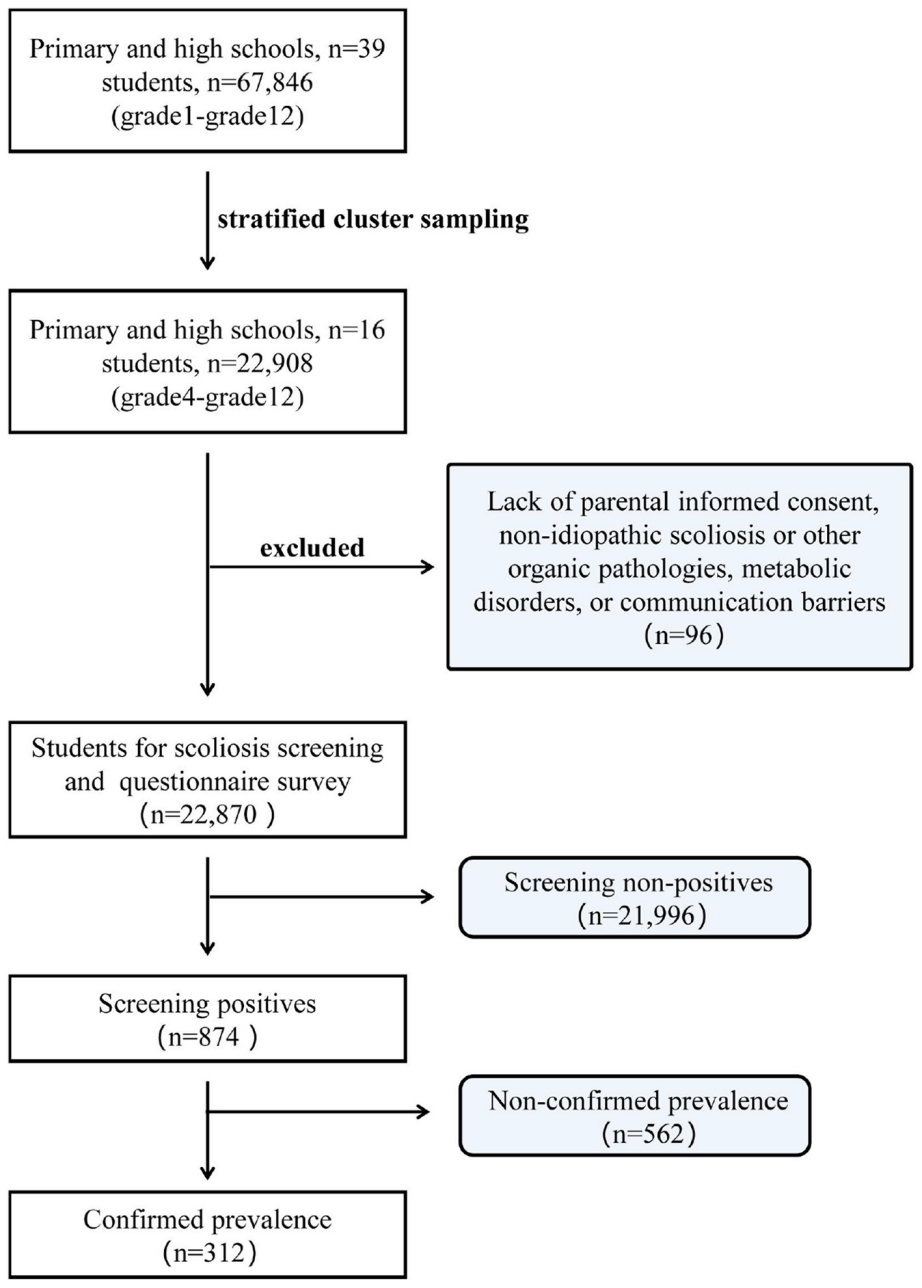
Flow diagram of AIS screening process and sample selection.

This study employed a cross-sectional design and was conducted between March and November 2022 in the Longlin Various Nationalities Autonomous County in Southwestern China. To balance sample representativeness and practical feasibility, this study adopted a stratified cluster sampling approach. Initially, all 39 schools in the county were stratified according to educational stage (primary, junior high, and senior high schools) and geographical location (county/town, village). Following this, schools were randomly selected from each stratum (Supplementary Table S1). Subsequently, all eligible students from grade 4 (primary school) to grade 12 (high school), aged 10 to 18 years, from 16 selected primary and secondary schools in the county were recruited as study participants. During the screening phase, 0.42% (*n* = 96) of the initially eligible participants were excluded. Exclusions were based on the following criteria: lack of parental informed consent; diagnosis of non-idiopathic scoliosis or other organic pathologies, such as congenital malformations, osteochondrodystrophy, or metabolic disorders, as confirmed by three senior spine surgeons; or the presence of communication barriers that could compromise examination validity (Figure 1). Missing data were evaluated using Little's MCAR test (*p* = 0.32), which indicated that values were missing completely at random. Given the low proportion of missing observations and the absence of systematic bias, a complete case analysis was employed, resulting in the exclusion of incomplete records. The final analytical sample included 22,814 students.

### 2.2 Scoliosis screening workflow

A dedicated screening team, comprising 20 examiners (all certified orthopedic surgeons or rehabilitation therapists with over 3 years of clinical experience), conducted the examinations. Scoliosis screening was integrated with the school's annual health check-up. The procedure strictly adhered to the *Guideline for adolescent scoliosis screening in China* (version 2024) and the national standard *Screening of spinal curvature abnormality of children and adolescents* (GB/T 16133-2014, China) (30). Participants were assessed by examiners of the same gender. To ensure diagnostic consistency, each child was independently evaluated by two examiners according to a standardized protocol. Any discrepancies between their assessments were adjudicated by a senior spine surgeon to establish the final diagnosis. Screening was conducted at schools by a uniformly trained screening team and included collection of basic information and anthropometric measurements, physical examination, and measurement of the angle of trunk rotation (ATR). Basic information such as age, gender, and ethnicity was collected. Height and weight were measured by trained personnel using calibrated instruments (each measured three times with the average taken, height accurate to 0.1 cm, weight accurate to 0.1 kg). BMI was subsequently calculated and categorized into underweight (BMI < 18.5), normal (18.5 ≤ BMI < 24), overweight (24 ≤ BMI < 28), and obese (BMI ≥ 28) according to the *National student physical health standard* (2014 revision, China).

To manage the large cohort efficiently, students were scheduled in batches. The average daily screening capacity was approximately 150–200 students. Participants progressed through a standardized sequence of assessments, beginning with registration and basic anthropometric measurement, followed by the Adams Forward Bend Test, visual and instrumental pelvic assessment, and spinal motion assessment. An ATR measurement was performed when clinically indicated, prior to final on-site data recording and verification.

### 2.3 Scoliosis examination procedure

The screening protocol comprised a sequential physical examination followed by radiographic confirmation for positive cases. The physical examination began with visual inspection of the standing posture for truncal asymmetry, followed by the Adams Forward Bend Test. In this test, participants stood with feet shoulder-width apart, knees fully extended, and bent forward slowly at the hips until the trunk was approximately parallel to the floor, with arms hanging relaxed and palms opposed. This maneuver facilitates the observation of a fixed rib hump or lumbar para-spinal prominence, serving to differentiate structural scoliosis, where asymmetry persists, from non-structural causes, where it may diminish with postural change. For those with a positive Adams forward bend test, a scoliometer was used to measure the ATR at the point of most significant back asymmetry, and the maximum value was recorded (31).

Pelvic alignment was assessed in the standing position via visual inspection of the iliac crests and quantified with a pelvic inclinometer on the posterior superior iliac spines, recording an asymmetry greater than 5°. Active spinal motions (forward bending, lateral bending, axial rotation) were also observed to assess flexibility and dynamic asymmetry.

A positive screening result was defined as meeting any one of the following criteria: positive visual inspection, positive Adams forward bend test, or ATR ≥5° (10). Subsequently, all screening positives were referred to the collaborating hospital for full-spine posteroanterior and lateral radiographs. Cobb angle in the coronal plane was measured by radiologists, and individuals with a Cobb angle ≥10° were ultimately diagnosed with scoliosis.

### 2.4 Questionnaire survey

To explore relevant influencing factors, a structured questionnaire was designed based on a review of domestic and international literature (32–34) and was pre-tested for clarity and comprehension in a pilot study. The questionnaire content covered demographic characteristics (e.g., age, gender, ethnicity, height, weight, BMI), family history (family history of scoliosis, frequency of posture reminders from guardians), learning-related factors [weekly physical education class frequency, backpack type, daily exercise types, reading/writing posture, desk/chair height adjustment (refers to adjustable classroom furniture)], and lifestyle habits (daily electronic device usage time, weekly outdoor activity time, daily sleep duration, sleep posture, engagement in physical labor, frequency of sweet food consumption). All variables were defined by well-defined, unambiguous survey questions to facilitate clear determination. For instance, the variable “desk/chair height adjustment” was assessed by a direct student self-report question: “Can the height of the desk and chair you use at school be adjusted to suit your height?”, with binary (Yes/No) response options. All questionnaire items were designed to be specific and closed-ended to minimize subjective interpretation. To mitigate recall bias and improve accuracy, the questionnaires were administered in a controlled classroom setting under the supervision of trained staff, who provided standardized instructions and were available to clarify questions. Students completed the questionnaires concurrently with the spinal screening to contextualize the questions within the health assessment framework.

### 2.5 Quality control

Strict quality control measures were implemented to ensure the reliability and accuracy of the research data, and to minimize inter-rater variability among all the examiners across the extensive screening period. Firstly, a screening team consisting of professional doctors and therapists from the Rehabilitation Medicine Department of Longlin Traditional Chinese Medicine Hospital was established. All staff involved in physical examinations and questionnaire administration received uniform standardized training and assessment to ensure consistency in the screening process and judgment criteria. This included theoretical review of the screening protocol, practical demonstrations, and hands-on practice sessions on examiners to standardize assessment techniques and interpretation of findings.

During screening, individuals with positive screening results required re-examination by a second physician; a case was only considered suspected if both deemed it positive, thereby reducing subjective error. Besides, senior research team members periodically observed and supervised screening sessions across different schools and time points to ensure ongoing adherence to the protocol. Brief re-calibration sessions were conducted monthly to address any procedural drift.

For data management, questionnaire data were double-entered by dedicated personnel, with a random sample of 5% of all completed screening forms was selected daily for cross-verification by a different examiner on-site for accuracy to maximize data entry correctness.

### 2.6 Ethical considerations

Parents, students, and teachers were informed about the purpose of the study and the details of the examination. Informed consent was obtained from all participating students and their parents, with signed consent forms. Given the multi-ethnic composition of the population (including Zhuang, Yi, and GeLao groups) and potential language barriers, the informed consent process was carefully adapted. Consent forms and participant information sheets were professionally translated and provided in both Mandarin Chinese and the predominant local minority languages. Bilingual research staff were present during consent sessions at each school to explain the study's purpose, procedures, risks, and benefits, and to answer any questions in the parent's preferred language, ensuring comprehensive understanding prior to signing. The study protocol has been granted administrative approval by the Longlin County Health Bureau (Approval No. 2021-2) and has been approved by the Longlin County Traditional Chinese Medicine Hospital Ethics Committee (Approval No. K2019-090-02). The guidelines, regulations, and principles of the Declaration of Helsinki were strictly followed.

### 2.7 Statistical analysis

Data organization was performed using Microsoft Office Excel 2007, and data analysis was conducted using SPSS 22.0 statistical software. Categorical data were described using frequencies (*n*) and percentages (%), and group comparisons were made using the χ^2^ test. To identify factors independently associated with scoliosis, we adopted a comprehensive modeling strategy. First, univariate analyses were performed on all 16 candidate variables. Variables showing statistical significance (*p* < 0.05) in the univariate analysis, along with a priori confounders (gender, age, and family history of scoliosis) deemed clinically relevant. To account for the potential clustering of students within schools, we estimated robust standard errors, clustering at the school level. Multicollinearity among the independent variables was assessed using the variance inflation factor (VIF), with a mean VIF of 1.85 indicating no substantial multicollinearity (all VIFs < 3.0; see Supplementary Table S2). The results are presented as adjusted odds ratios (aOR) with 95% confidence intervals (CI). A *p*-value < 0.05 was considered statistically significant for all analyses.

## 3 Results

### 3.1 AIS screening and severity distribution

A total of 22,814 adolescents aged 10 to 18 years were screened in this study, including 12,149 boys and 10,665 girls. There were 874 screening positives, resulting in an overall positive rate of 3.83%. Following radiographic confirmation, 312 AIS cases were diagnosed, yielding a total prevalence of 1.37%. The positive predictive value of the screening was 35.70% (Supplementary Table S3).

#### 3.1.1 Gender, ethnicity, and age screening

Screening results showed significant variations across gender, ethnicity, and age groups (Supplementary Table S3). The screening positive rate was significantly higher in girls than in boys (4.64% vs. 3.12%, χ^2^ = 50.67, *p* < 0.001). However, the difference in the confirmed prevalence rates between genders was not statistically significant (1.47% in girls vs. 1.28% in boys, χ^2^ = 1.42, *p* = 0.233). Notably, the positive predictive value (PPV) (i.e., confirmed prevalence-to-screening positive ratio) was substantially higher among screening positive boys (40.90%) than girls (31.72%).

**Figure 2:**
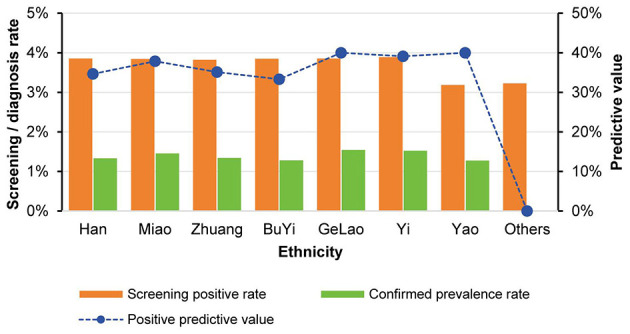
Trends in screening positive rates and confirmed AIS prevalence rates across different ethnic groups.

While no statistically significant difference was observed in the screening positive rate among students of different ethnicities (χ^2^ = 12.79, *p* = 0.078), the confirmed prevalence rate varied significantly across ethnic groups by the statistical test (χ^2^ = 15.62, *p* = 0.029). It should be noted, however, that the numerical prevalence was relatively higher in Yi (1.52%) and GeLao (1.54%) adolescents than in Han (1.33%) and Yao (1.27%) adolescents (Supplementary Table S3). In terms of screening accuracy, the Yi ethnic group demonstrated a PPV of 39.13%, while the GeLao and Yao ethnic groups both reached a PPV of 40.00% (Figure 2). However, it is important to note that the small number of cases in these and other minority groups (e.g., BuYi, Yao) results in wide confidence intervals that include values comparable to the overall prevalence, limiting firm conclusions about ethnic-specific differences at this stage (Supplementary Table S3).

**Figure 3:**
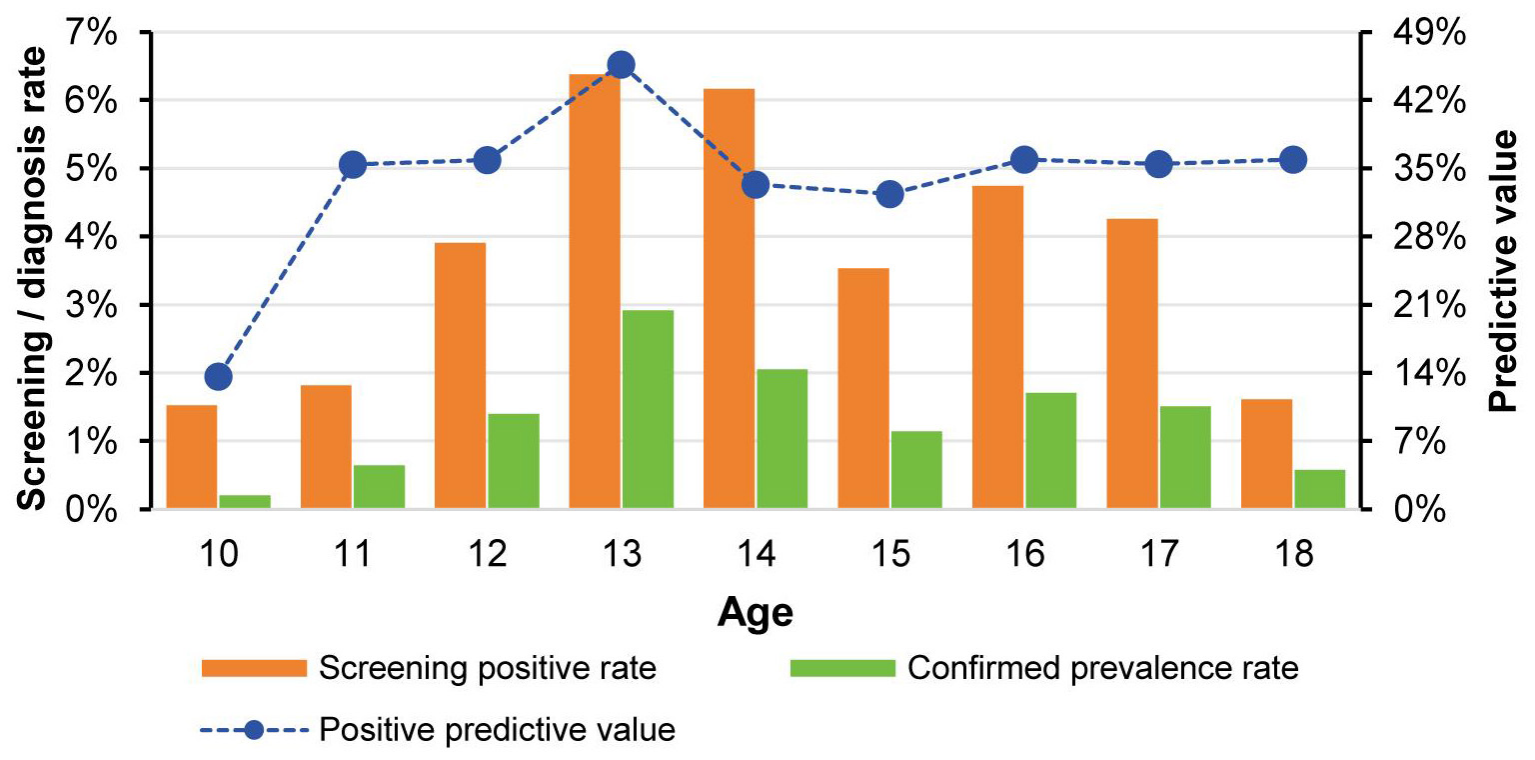
Trends in screening positive rates and confirmed AIS prevalence rates across different age groups.

Both the screening positive rate and the confirmed prevalence rate across age groups demonstrated a trend of initial increase followed by a decrease, peaking in the 13-year-old group (screening positive rate: 6.38%; confirmed prevalence rate: 2.92%) and reaching the lowest point in the 10-year-old group (screening positive rate: 1.52%; confirmed prevalence rate: 0.21%) (Figure 3). The PPV showed considerable variation across age groups, ranging from 13.64% in the 10-year-old group to 45.69% in the 13-year-old group, indicating substantially higher screening accuracy in the latter group.

#### 3.1.2 Cobb angle severity of gender and ethnicity

Among the 312 confirmed patients, the numbers of male and female patients were roughly equivalent (155 males, 49.68%; 157 females, 50.32%). Analysis of Cobb angle severity showed that male patients were predominantly in the mild scoliosis category (10°- < 20°) (89.68%), while 80.89% of female patients had mild scoliosis, with proportions of moderate and severe cases slightly higher than in males. However, Fisher's exact test indicated that the distribution of Cobb angle severity among patients of different genders was not statistically significant (*p* = 0.071) (Table 1).

**Table 1:** Distribution of Cobb angle severity [*n* (%)] among scoliosis patients by gender and ethnicity.

**Variable**	**Confirmed cases (*n*)**	**Cobb angle**		
		**Mild 10**°**–**<**20**°	**Moderate 20**°**–**<**30**°	**Severe** ≥**30**°
**Gender**				
Boy	155	139 (89.68%)	14 (9.03%)	2 (1.29%)
Girl	157	127 (80.89%)	28 (17.83%)	2 (1.27%)
**Ethnicity**				
Han	75	66 (88.00%)	8 (10.67%)	1 (1.33%)
Miao	64	57 (89.06%)	6 (9.38%)	1 (1.56%)
Zhuang	150	123 (82.00%)	25 (16.67%)	2 (1.33%)
BuYi	1	1 (100.00%)	0 (0.00%)	0 (0.00%)
GeLao	7	7 (100.00%)	0 (0.00%)	0 (0.00%)
Yi	13	12 (92.31%)	1 (7.69%)	0 (0.00%)
Yao	2	2 (100.00%)	0 (0.00%)	0 (0.00%)
Total	312	266 (85.26%)	42 (13.46%)	4 (1.28%)

Regarding ethnic composition, the Zhuang ethnicity had the highest number of confirmed cases (150 cases, 48.08%), followed by the Han (75 cases, 24.04%) and Miao (64 cases, 20.51%) ethnicities. The Cobb angle distribution for patients across all ethnic groups was predominantly mild. All confirmed cases from the BuYi, GeLao, and Yao ethnic groups were mild. The distribution of Cobb angle severity among confirmed patients of different ethnicities showed no statistically significant difference (*p* = 0.829). Overall, among the AIS patients confirmed in this study, 85.26% (266/312) were mild, 13.46% (42/312) were moderate, and only 1.28% (4/312) were severe (Table 1).

### 3.2 Analysis of influencing factors of scoliosis

#### 3.2.1 Univariate analysis

Univariate analysis of the 16 potential associated factors revealed that nine factors were significantly associated with the occurrence of scoliosis (all *p* < 0.05). These included BMI, desk/chair height adjustment, nighttime sleep posture, frequent sweet consumption, weekly physical education (PE) class frequency, maintaining correct reading/writing posture, total daily electronic device usage time, weekly outdoor activity time, and daily sleep duration. The remaining seven factors (gender, age, family history of scoliosis, backpack type, daily exercise type, engagement in physical labor) showed no significant influence (all *p* > 0.05) (Table 2).

**Table 2:** Univariate analysis of associated factors for scoliosis.

**Variable**	**Category**	**Total (*n*)**	**Scoliosis cases (*n*)**	**Prevalence**	**χ^2^ value**	***p*-value**
Gender	Boy	12,149	155	1.28%	1.42	0.233
	Girl	10,665	157	1.47%		
Age (years)	≤ 14	10,591	154	1.45%	1.17	0.28
	>14	12,223	158	1.29%		
BMI	Underweight	613	43	7.01%	158.6	< 0.001
	Normal	22,004	264	1.20%		
	Overweight	51	2	3.92%		
	Obese	146	3	2.05%		
Family history of scoliosis	Yes	419	5	1.19%	0.11	0.738
	No	22,395	307	1.37%		
Backpack type	Single-shoulder	1,056	15	1.42%	0.05	0.82
	Backpack	21,758	297	1.37%		
Daily exercise type	Unilateral sports	350	6	1.71%	0.31	0.581
	Bilateral sports	22,464	306	1.36%		
Desk/chair height adjustment	No	450	27	6.00%	72.8	< 0.001
	Yes	22,364	285	1.27%		
Nighttime sleep posture	Supine	5,000	35	0.70%	24.6	< 0.001
	Lateral	9,000	157	1.74%		
	Variable	8,814	120	1.36%		
Engagement in physical labor	No	17,856	245	1.37%	0.04	0.838
	Yes	4,958	67	1.35%		
Frequent sweet consumption	Yes	11,932	185	1.55%	6.19	0.013
	No	10,882	127	1.17%		
Weekly PE class frequency	0 times	500	22	4.40%	40.9	< 0.001
	1 time	8,952	96	1.07%		
	2 times	13,362	194	1.45%		
Maintaining correct reading/writing posture	No	986	35	3.55%	105.5	< 0.001
	Occasionally	1,975	66	3.34%		
	Always	19,853	211	1.06%		
Total daily electronic device time (h)	< 1 h	9,973	50	0.50%	45.2	< 0.001
	1–2 h	7,965	158	1.98%		
	>2 h	4,876	94	1.93%		
Weekly outdoor activity time (h)	< 1 h	4,982	82	1.65%	20.1	< 0.001
	1–3 h	9,976	160	1.60%		
	>3 h	7,856	70	0.89%		
Daily sleep time (h)	< 6 h	4,968	61	1.23%	17	< 0.001
	6–8 h	7,893	142	1.80%		
	>8 h	9,953	109	1.09%		

Specifically, adolescents who were underweight had the highest prevalence rate (7.01%), significantly higher than those in the normal weight, overweight, and obese groups (χ^2^ = 158.6, *p* < 0.001). The prevalence rate among those without adjusted desk/chair height (6.00%) was significantly higher than in the adjusted group (1.27%) (χ^2^ = 72.8, *p* < 0.001). Regarding sleep posture, those who slept in a lateral position had a higher prevalence rate (1.74%) than those who slept supine (0.70%) or changed positions (1.36%) (χ^2^ = 24.6, *p* < 0.001). Furthermore, lack of physical exercise (prevalence 4.40% in the group with 0 weekly PE classes), poor reading/writing posture (prevalence 3.55% in the group not maintaining correct posture), daily electronic device use exceeding 1 h, insufficient weekly outdoor activity, and daily sleep duration of 6–8 h were all associated with higher risk (Table 2).

#### 3.2.2 Multivariate logistic regression analysis

To further identify independent risk factors, the nine variables that were significant in the univariate analysis were included in a multivariate logistic regression model. The results showed that abnormal BMI (especially underweight and overweight), lack of desk/chair height adjustment, poor sleep posture (lateral and variable positions), lack of physical exercise (0 weekly PE classes), poor reading/writing habits, daily electronic device use time >1 h, insufficient outdoor activity, abnormal sleep duration (6–8 h), and frequent sweet consumption were all independently associated with adolescent scoliosis (all *p* < 0.05) (Tables 3 and Supplementary Table S4).

**Table 3:** Multivariate logistic regression analysis of associated factors for scoliosis.

**Independent variable**	**Variable category**	**β value**	**SE**	**Wald χ^2^ value**	***P*-value**	**aOR**	**95% CI**
BMI	Normal					1	1.0
	Underweight	1.609	0.227	50	< 0.001	5	3.197.81
	Overweight	0.96	0.45	4.57	0.032	2.61	1.08–6.30
	Obese	0.511	0.362	2	0.157	1.67	0.80–3.48
Nighttime sleep posture	Supine					1	1.0
	Lateral	0.788	0.159	24.6	0.011	2.2	1.61–3.00
	Variable	0.405	0.16	6.4	< 0.001	1.5	1.09–2.07
Desk/chair height adjustment	Yes					1	1.0
	No	1.386	0.164	72.8	< 0.001	4	2.90–5.51
Frequent sweet consumption	No					1	1.0
	Yes	0.262	0.105	6.19	0.013	1.3	1.06–1.59
Weekly PE class frequency	2 times					1	1.0
	1 time	−0.223	0.158	2	0.157	0.8	0.59–1.09
	0 times	1.029	0.161	40.9	< 0.001	2.8	2.04–3.84
Maintaining correct reading/writing posture	Always					1	1.0
	Occasionally	0.916	0.167	30	< 0.001	2.5	1.80–3.46
	No	1.098	0.107	105.5	< 0.001	3	2.43–3.69
Total daily electronic device time (h)	< 1 h					1	1.0
	1–2 h	1.253	0.186	45.2	< 0.001	3.5	2.43–5.05
	>2 h	1.098	0.199	30	< 0.001	3	2.03–4.43
Weekly outdoor activity time (h)	>3 h					1	1.0
	1–3 h	0.531	0.137	15	0.001	1.7	1.30–2.23
	< 1 h	0.587	0.131	20.1	< 0.001	1.8	1.39–2.33
Daily sleep time (h)	>8 h					1	1.0
	6–8 h	0.47	0.114	17	0.001	1.6	1.28–2.00
	< 6 h	0.095	0.134	0.5	0.48	1.1	0.85–1.43

Specifically, compared to those with normal BMI, the association of developing scoliosis increased to 5.00 times (95% CI: 3.19–7.81) for underweight adolescents and 2.61 times (95% CI: 1.08–6.30) for overweight adolescents. The association for those without desk/chair height adjustment was 4.0 times (95% CI: 2.90–5.51) that of those with adjustment. Regarding sleep posture, the association for those sleeping in a lateral position and those with variable positions was 2.2 times (95% CI: 1.61–3.00) and 1.5 times (95% CI: 1.09–2.07) that of those sleeping supine, respectively. Adolescents with no weekly PE classes had a 2.8 times higher association (95% CI: 2.04–3.84) compared to those with 2 classes per week. Concerning reading/writing posture, the risk for those who did not maintain and those who occasionally maintained correct posture was 3.0 times (95% CI: 2.43–3.69) and 2.50 times (95% CI: 1.80–3.46) that of those who always maintained correct posture, respectively. Furthermore, the association for the groups using electronic devices for 1–2 h and >2 h daily was 3.5 times and 3.0 times that of the group using them for < 1 h, respectively (Table 3).

## 4 Discussion

This study represents a large-scale screening for AIS among adolescents aged 10–18 years in a multi-ethnic region in Southwestern China, systematically analyzing its prevalence, demographic characteristics, and key data from the school screening process. Our multivariate analysis identified a cluster of modifiable lifestyle and behavioral factors as independent predictors for AIS. The results not only provide valuable first-hand data on the spinal health status of adolescents in this region but also offer new insights and directions for thought regarding the epidemiological characteristics of AIS, screening strategies, and potential etiological mechanisms.

### 4.1 Overall prevalence analysis

This study found the overall prevalence of AIS in this region to be 1.37%, a result largely consistent with reported prevalence ranges globally and in Asia. For instance, a systematic review reported a global AIS prevalence of 1.34%, while studies in Asia report figures between 0.4% and 2.5% (6), and the prevalence among 10- to 16-year-olds in the United States is approximately 1%−3% (10). Our data align with these macro-level figures, indicating that despite regional and ethnic variations, AIS, as a common skeletal development issue in adolescents, exhibits a certain universality in its incidence.

Furthermore, this study observed that the AIS prevalence peaked at 13–14 years of age, highly correlating with the adolescent PHV period (9). During PHV, both the biomechanical load on the spine and its growth rate reach their peak, which is considered a critical window for the onset and progression of AIS. A previous large-scale study of individuals aged 4–20 years in China also showed that the peak incidence of AIS was concentrated between 13 and 15 years (12). The findings of this study further corroborate the scientific basis and necessity of focusing school screening efforts on adolescents aged 10–15, particularly those aged 13–14.

### 4.2 The impact of ethnic differences on AIS

This study identified differences in AIS screening outcomes among students of different ethnicities. It is noteworthy that these disparities were primarily observed at the confirmation stage rather than during screening. Specific data revealed that students of Yi (1.52%) and GeLao (1.54%) ethnicity demonstrated relatively higher AIS confirmation rates, while Han (1.33%) and Yao (1.27%) students showed comparatively lower rates. While our study reveals intriguing ethnic variations, the relatively small sample sizes for some minority groups (e.g., BuYi, Yi) necessitate cautious interpretation and call for validation in larger, multi-center studies. This observation aligns with trends noted in previous research (19, 20, 22, 35–38), suggesting that the epidemiological characteristics of AIS may vary among different ethnic populations. However, these differences likely reflect the complex interplay of multiple factors rather than being determined solely by ethnicity.

The observed ethnic variations suggest the potential involvement of a complex interplay of factors. While genetic predisposition is postulated to play a role in AIS based on the existing literature (39–41), our study was not designed to identify specific genetic determinants. The differences we observed may be more directly attributable to, or confounded by, varying environmental conditions, nutritional status, and lifestyle behaviors across ethnic groups (42–44). Factors such as the intake of specific micronutrients, vitamin D levels, and participation in particular types of physical activity may indirectly affect the occurrence and progression of AIS by influencing bone development and biomechanical balance (45).

Therefore, future research in similar multi-ethnic regions should employ more representative samples and refined study designs to systematically evaluate the complex relationships between genetic, environmental, and lifestyle factors. This approach will enable a more comprehensive understanding of the etiological characteristics of AIS and provide scientific evidence for developing targeted prevention strategies.

### 4.3 Atypical manifestation of gender differences

Although numerous literatures report a significantly higher prevalence of AIS in females than in males (12, 17, 18, 46, 47), this study found no statistical difference in the confirmed prevalence between girls (1.47%) and boys (1.28%). However, during the screening process, the screening positive rate was significantly higher in girls (4.64%) than in boys (3.12%), while PPV was much higher in boys (40.90%) than in girls (31.72%). This phenomenon reveals that gender may influence the AIS screening and diagnosis process in more complex ways.

The higher screening positive rate coupled with lower PPV in girls invites several non-mutually exclusive hypotheses. Firstly, biological factors specific to female adolescence may contribute. It has been postulated that hormonal fluctuations, particularly in estrogen levels, could temporarily affect spinal tissue compliance and postural control during the peripubertal growth spurt (48, 49). This might lead to observable asymmetries in the forward bend test that do not yet correspond to a fixed structural curvature meeting the Cobb angle criterion (50, 51). Secondly, gender differences in body composition, with girls typically having a higher fat-to-muscle ratio, might influence the biomechanical presentation and the sensitivity of the Adams test (52, 53). Furthermore, we explored other potential contributing factors. The age distribution of our cohort was similar between genders, making this an unlikely sole explanation. However, the screening criteria themselves, which are highly sensitive, might interact differently with the female physiology described above. Sociocultural factors, such as heightened body awareness leading to earlier care-seeking or more cautious examiner assessment, could also contribute to the initial higher positive rate, though this study was not designed to test this specifically.

Conversely, the higher PPV in boys suggests that when a positive screen occurs, it is more likely to represent true AIS. This could be explained if stronger para-spinal muscles in males conceal milder curves, meaning that screening tends to detect only the more pronounced deformities in boys. The biological mechanism behind this phenomenon remains unclear, but it reminds us that when interpreting screening results and formulating referral strategies, gender specificity should be fully considered to avoid neglecting attention to boys due to the inherent notion of “higher girl incidence.”

### 4.4 Significant associated factors for adolescent scoliosis

The results of this study indicate that abnormal BMI, poor postural habits, lack of exercise, excessive electronic device use, and irregular routines are the main associated factors for the development of scoliosis in adolescents, while cultivating healthy lifestyle habits and exercise patterns may help reduce the risk. This finding aligns with several international studies, emphasizing the important role of multi-dimensional lifestyle factors in AIS onset. Firstly, the association between abnormal BMI (especially low BMI) and AIS has been confirmed in previous research (54, 55). Low BMI may reflect poor nutritional status or weak bone and muscle development, thereby reducing spinal stability and increasing the risk of curvature (56). Low body weight (often indicated by a low BMI) may predispose adolescents to spinal curvature due to diminished para-spinal muscular mass and lower bone mineral density. Conversely, it is also plausible that progressive scoliosis contributes to weight loss or a failure to gain weight appropriately (57). Secondly, poor postural habits (such as incorrect reading/writing posture, sitting with crossed legs, inappropriate desk height) have been associated with spinal deformity through asymmetric biomechanical loading (58, 59). For instance, maintaining a forward-leaning standing posture or consistently sleeping on one side may exacerbate asymmetric stress distribution on the spine (59). Thirdly, the association between lack of exercise and AIS may stem from insufficient core muscle strength, unable to provide adequate support for the spine (60). It is noteworthy that the type of exercise is also crucial; certain high-risk sports (like ballet) may increase the risk due to repetitive asymmetric movements (44, 54), while regular moderate-intensity exercise has a protective effect (60). Fourthly, excessive electronic device use (daily screen time >2 h) not only directly prolongs the duration of maintaining static poor postures but may also displace physical activity time, creating a vicious cycle (58, 61). Finally, irregular routines (such as insufficient sleep in this study) may be related to impaired muscle fatigue recovery and hormonal secretion disorders (e.g., abnormal rhythms of growth hormone and melatonin), indirectly affecting spinal health (56, 62). Moreover, sleep insufficiency (6–8 h) was associated with higher odds of AIS compared to the recommended duration (>8 h). Although typical guidelines recommend 8–10 h, empirical data from our study indicate that a substantial majority of adolescents, likely due to academic pressures and substantial homework loads, consistently achieve less than 8 h of sleep (63). In summary, AIS prevention requires comprehensive strategies, including nutritional intervention [e.g., increasing dairy intake (58)], postural correction education, exercise guidance (emphasizing symmetric exercises and core muscle training), and screen time management. Future research should focus on the interactions among these modifiable factors to provide a basis for developing precise public health intervention measures. However, as a cross-sectional study, the associations observed here do not imply causation, and the temporal sequence between these factors and AIS onset cannot be determined.

### 4.5 Consideration of reverse causality and unmeasured confounding

It is crucial to consider alternative explanations for the observed associations, particularly reverse causality. For instance, adolescents with subclinical or undiagnosed spinal deformity might avoid physical activities due to discomfort or self-consciousness, leading to physical inactivity and potentially lower BMI, rather than these factors causing the scoliosis. Similarly, excessive screen time could be a consequence of social withdrawal related to postural concerns. While we adjusted for key demographic and clinical variables, the potential for residual confounding by unmeasured factors, such as socioeconomic status, parental education, and precise pubertal stage, remains. These factors could influence both lifestyle habits (e.g., nutrition, access to sports) and spinal health. Therefore, our findings should be interpreted as identifying important and modifiable correlates of AIS, which merit further investigation in longitudinal studies to elucidate their potential causal roles.

### 4.6 Optimization and challenges of school screening strategy

The total screening positive rate of 3.83% and the positive predictive value of 35.70% in this study highlight the significant value of school screening as the first line of defense for the early detection of AIS. Simultaneously, to optimize screening strategies, establishing an efficient “school-hospital” collaborative referral mechanism is crucial. This model helps ensure the quality of screening and the continuity of subsequent management. However, international experience shows that even with established screening programs, low referral compliance and loss to follow-up remain common challenges (64, 65). Therefore, a successful cooperation model requires not only a clear referral process but also health education for students and parents, psychological support, and a convenient follow-up appointment system to improve referral rates and treatment compliance (66). Especially in multi-ethnic, relatively remote areas like those involved in this study, establishing a tiered diagnosis and treatment network involving telemedicine consultations and primary healthcare institutions could be an effective way to overcome geographical and cultural barriers. Additionally, this study observed a certain proportion of false-positive results in the screening. Future efforts could explore a multi-stage screening process or incorporate more discriminative auxiliary tools (such as surface topography) to enhance screening specificity, while strengthening regular follow-up for high-risk groups to reduce the risk of false negatives.

### 4.7 Study limitations and future directions

This study reveals the prevalence characteristics of AIS in a multi-ethnic region of Southwestern China, providing new perspectives, particularly regarding ethnic and gender differences. These findings not only enrich our understanding of AIS epidemiology but also provide a solid scientific basis for optimizing regional school screening programs and formulating precise public health intervention strategies. Although this study has yielded some important findings, it still has certain limitations. Firstly, the sample sizes for some minority ethnic groups (such as BuYi, Yi) were relatively small, and the calculated prevalence rates might have some random error, requiring validation in larger populations. Secondly, the cross-sectional design precludes causal inference and necessitates caution in interpreting the direction of the observed associations, as reverse causality (e.g., AIS leading to reduced activity) is plausible. Furthermore, although we adjusted for several confounders, residual confounding by unmeasured variables such as socioeconomic status, parental education, and precise pubertal staging cannot be ruled out. For example, we cannot determine whether girls who were initially screen-positive but not confirmed will develop AIS in the future. Thirdly, we acknowledge that engagement could be influenced by variations in health literacy and communication channels among parents. To address this, future studies will implement more tailored, community-based strategies to strengthen inclusivity and ensure equitable representation across all groups.

Based on the findings and limitations of this study, future research could be developed in the following directions. First, a long-term adolescent cohort should be established, with particular emphasis on regularly following up students who screened positive initially but were not diagnosed. Specifically, as a key component of comprehensive school screening strategies, these individuals should undergo periodic clinical monitoring until skeletal maturity. This approach would help track the progression of spinal morphology and further investigate the roles of hormonal levels, biomechanical parameters, and lifestyle in the onset and development of AIS. Second, prospective studies conducted in identified high-risk groups would be valuable to assess the effectiveness of early non-surgical interventions, including scoliosis-specific exercises and postural correction guidance, in preventing the onset or slowing the progression of AIS. Third, future studies should explore the integration of advanced screening and assessment tools, such as 3D ultrasound and deep learning technologies (67, 68). These innovative approaches show promise in enhancing the objectivity, efficiency, and accessibility of screening, while providing more accurate quantification of spinal three-dimensional morphology and progression risk. Fourth, the measurement of lifestyle factors relied on self-reported questionnaires, which are susceptible to recall and misclassification biases. Students might inaccurately recall certain behaviors (e.g., exact screen time) or provide socially desirable answers. Although we implemented measures such as questionnaire pre-testing and standardized administration to enhance data quality, the potential for non-differential misclassification remains. Such misclassification would typically bias the observed associations toward the null, suggesting that the true strengths of the associations we identified might be even stronger than reported.

## 5 Conclusion

This large-scale study establishes a 1.37% prevalence of AIS among multi-ethnic adolescents in Southwestern China, which is consistent with global figures. It reveals notable variations in prevalence across ethnic groups and a distinct gender-specific pattern in screening accuracy, with girls showing a higher false-positive rate. Importantly, we observed several modifiable factors associated with AIS, including abnormal BMI, poor postural habits, physical inactivity, prolonged electronic device use, and inadequate sleep. These findings underscore the necessity of implementing tailored school-screening programs and integrating multifaceted public health interventions targeting these associated factors to mitigate the burden of AIS in this and similar populations.

## Data Availability

The original contributions presented in the study are included in the article/Supplementary material, further inquiries can be directed to the corresponding author.
