# Supplementary material for: Prevalence and associated factors of multi-ethnic adolescent idiopathic scoliosis in Longlin, Southwestern China: a school-based cross-sectional study
Source: Front Public Health. 2025 Dec 15;13:1729509. doi: 10.3389/fpubh.2025.1729509 (PMC12745271; doi:10.3389/fpubh.2025.1729509)
Supplement: Supplementary file 1 [file Supplementary_file_1.docx]

***Supplementary information***

Table S1 Stratified cluster sampling of schools in **Longlin**

| **Educational stage** | **Total** | | **Stratified cluster sampling** | | **Grade level** |
| --- | --- | --- | --- | --- | --- |
|  | **No. of schools in county/town** | **No. of schools in village** | **No. of schools in county/town** | **No. of schools in village** |  |
| Primary school | 4 | 17 | 2 | 6 | grade 4 -grade 6 |
| Junior high school | 16 | 0 | 5 | 0 | grade 7 -grade 9 |
| Senior high school | 3 | 0 | 3 | 0 | grade 10 -grade 12 |

Table S2. Assessment of multicollinearity using Variance Inflation Factor (VIF) in the multivariable logistic regression model

| **Variable** | **VIF** |
| --- | --- |
| Sex (Girl) | 1.12 |
| Gender (>14 years) | 1.31 |
| Family History of Scoliosis (Yes) | 1.02 |
| BMI (Underweight) | 1.18 |
| BMI (Overweight) | 1.09 |
| BMI (Obese) | 1.07 |
| Desk/Chair Height Adjustment (No) | 1.15 |
| Nighttime Sleep Posture (Lateral) | 1.24 |
| Nighttime Sleep Posture (Variable) | 1.23 |
| Frequent Sweet Consumption (Yes) | 1.11 |
| Weekly PE Class Frequency (0 times) | 1.33 |
| Weekly PE Class Frequency (1 time) | 1.29 |
| Maintaining Correct Posture (Occasionally) | 1.42 |
| Maintaining Correct Posture (No) | 1.51 |
| Daily Electronic Device Time (1~2h) | 1.38 |
| Daily Electronic Device Time (>2h) | 1.41 |
| Weekly Outdoor Activity Time (1~3h) | 1.35 |
| Weekly Outdoor Activity Time (<1h) | 1.32 |
| Daily Sleep Time (6~8h) | 1.27 |
| Daily Sleep Time (<6h) | 1.26 |
| **Mean VIF** | **1.85** |

**Table S3. Prevalence of AIS according to gender, ethnicity, and age**

| **Variable** | **Number** | **Primary screening rate** | | **Confirmed prevalence rate** | | **positive predictive value** |
| --- | --- | --- | --- | --- | --- | --- |
|  |  | **Number** | **Rate** | **Number** | **Rate (95%CI)** |  |
| **Gender** | | | | | | |
| boy | 12149 | 379 | 3.12% | 155 | 1.28% (1.09, 1.50) | 40.90% |
| girl | 10665 | 495 | 4.64% | 157 | 1.47% (1.26, 1.72 | 31.72% |
| **Ethnicity** | | | | | | |
| Han | 5998 | 231 | 3.85% | 80 | 1.33% (1.06, 1.67) | 34.63% |
| Miao | 4399 | 169 | 3.84% | 64 | 1.45% (1.14, 1.85) | 37.87% |
| Zhuang | 11171 | 427 | 3.82% | 150 | 1.34% (1.14, 1.57) | 35.13% |
| BuYi | 78 | 3 | 3.85% | 1 | 1.28% (0.23, 7.02) | 33.33% |
| GeLao | 389 | 15 | 3.86% | 6 | 1.54% **(0.74, 3.21)** | 40.00% |
| Yi | 591 | 23 | 3.89% | 9 | 1.52% **(0.88, 2.63)** | 39.13% |
| Yao | 157 | 5 | 3.18% | 2 | 1.27% (0.32, 5.02) | 40.00% |
| Others* | 31 | 1 | 3.23% | 0 | 0.00% (0.00, 11.2) | 0.00% |
| **Age (years)** | | | | | | |
| 10 | 1447 | 22 | 1.52% | 3 | 0.21% (0.07, 0.61) | 13.64% |
| 11 | 2637 | 48 | 1.82% | 17 | 0.64% (0.40, 1.03) | 35.42% |
| 12 | 2353 | 92 | 3.91% | 33 | 1.40% (1.00, 1.96) | 35.87% |
| 13 | 1818 | 116 | 6.38% | 53 | 2.92% (2.23, 3.80) | 45.69% |
| 14 | 2336 | 144 | 6.16% | 48 | 2.05% (1.55, 2.72) | 33.33% |
| 15 | 2970 | 105 | 3.54% | 34 | 1.14% (0.82, 1.60) | 32.38% |
| 16 | 3519 | 167 | 4.75% | 60 | 1.71% (1.32, 2.19) | 35.93% |
| 17 | 3311 | 141 | 4.26% | 50 | 1.51% (1.14, 1.99) | 35.46% |
| 18 | 2423 | 39 | 1.61% | 14 | 0.58% (0.34, 0.97) | 35.90% |
| **Total** | **22814** | 874 | 3.83% | 312 | 1.37% **(1.23, 1.53)** | 35.70% |

* “Others” includes 7 Dong, 1 HaNi, 1 Hui, 2 Li, 3 Manchus, 3 MaoNan, 1 Shui, 11 TuJia, and 2 Dai.

**Table S4. Variable Assignment**

| **Variable** | **Category** | **Assignment** |
| --- | --- | --- |
| BMI | Underweight | 1 |
|  | Normal | 0 |
|  | Overweight | 2 |
|  | Obese | 3 |
| Desk/Chair Height Adjustment | No | 1 |
|  | Yes | 0 |
| Nighttime Sleep Posture | Supine | 0 |
|  | Lateral | 1 |
|  | Variable | 2 |
| Frequent Sweet Consumption | Yes | 1 |
|  | No | 0 |
| Weekly PE Class Frequency | 0 times | 2 |
|  | 1 time | 1 |
|  | 2 times | 0 |
| Maintaining Correct Reading/Writing Posture | No | 2 |
|  | Occasionally | 1 |
|  | Always | 0 |
| Total Daily Electronic Device Time (h) | <1h | 0 |
|  | 1~2h | 1 |
|  | >2h | 2 |
| Weekly Outdoor Activity Time (h) | <1h | 2 |
|  | 1~3h | 1 |
|  | >3h | 0 |
| Daily Sleep Time (h) | <6h | 2 |
|  | 6~8h | 1 |
|  | >8h | 0 |
